# Using Functional Annotation for the Empirical Determination of Bayes Factors for Genome-Wide Association Study Analysis

**DOI:** 10.1371/journal.pone.0014808

**Published:** 2011-04-27

**Authors:** Jo Knight, Michael R. Barnes, Gerome Breen, Michael E. Weale

**Affiliations:** 1 Department of Medical and Molecular Genetics, King's College London School of Medicine, London, United Kingdom; 2 National Institute for Health Research (NIHR), Biomedical Research Centre Guy's and St. Thomas' NHS Foundation Trust and King's College London, London, United Kingdom; 3 Computational Biology, GlaxoSmithKline, Stevenage, United Kingdom; 4 Social, Genetic and Developmental Psychiatry Centre, Institute of Psychiatry, King's College London School of Medicine, London, United Kingdom; Aarhus University, Denmark

## Abstract

A genome wide association study (GWAS) typically results in a few highly significant ‘hits’ and a much larger set of suggestive signals (‘near-hits’). The latter group are expected to be a mixture of true and false associations. One promising strategy to help separate these is to use functional annotations for prioritisation of variants for follow-up. A key task is to determine which annotations might prove most valuable. We address this question by examining the functional annotations of previously published GWAS hits. We explore three annotation categories: non-synonymous SNPs (nsSNPs), promoter SNPs and *cis* expression quantitative trait loci (eQTLs) in open chromatin regions. We demonstrate that GWAS hit SNPs are enriched for these three functional categories, and that it would be appropriate to provide a higher weighting for such SNPs when performing Bayesian association analyses. For GWAS studies, our analyses suggest the use of a Bayes Factor of about 4 for *cis* eQTL SNPs within regions of open chromatin, 3 for nsSNPs and 2 for promoter SNPs.

## Introduction

New clues about the aetiology of complex genetic diseases have been provided by genome-wide association studies (GWAS) [1]. Since SNPs across the genome are investigated in GWAS, this allows such studies to identify causal variants which may never have been previously suspected to be involved in the trait. Notwithstanding the advantages of this ‘hypothesis-free’ or ‘hypothesis -neutral’ approach, it has become clear that effect sizes of many of the common variants involved in complex diseases are so small that even very large GWAS do not have full power to detect them [2]. This leads to a situation where, while each GWAS may result in a small number of genome-wide significant hits (those for which p-values are low enough to distinguish from false associations that occur by chance), there are a large number of true hits hidden within the association signals with p-values that are suggestive but not conclusive of true association.

Several lines of evidence suggest that these near hits do indeed contain some real association signals. Firstly, quantile-quantile plots of GWAS association p-values often show a departure from null expectation that extends into the ranked SNPs below the genomewide significance threshold [3]. Secondly, various forms of pathway analysis have reported significant biological dependency between near hit SNPs [4]. Thirdly, and most directly, GWAS meta-analysis often finds new hits that only appeared as near hits in smaller GWASs [5], [6].

Prioritization of near hits for follow-up may be more effective if functional information is combined with the GWAS p-values. There is already evidence that causative SNPs for a wide range of traits are enriched for certain functional categories [7] [8] and an increasing amount of annotation is available that could be used for such studies. There are annotations relating to gene structure, predicted function of nsSNPs, regulatory regions, DNA structure and many more [9]. Various statistical methods are now available for the analysis of p-values that have been weighted according to some user-defined scheme [10], [11], [12], [13], [14]. However, a key aspect of all these studies is that they use subjective weighting schemes. In this study, we propose empirically derived weightings within a Bayesian framework.

One way to arrive at an objective, empirically based weighing scheme is to use the observed preponderance of functional annotations in established GWAS hits as a guide to weighting of ‘near hit’ GWAS SNPs. GWAS data are more appropriate for this purpose than candidate gene genotyping data, as the SNPs typed in the latter type of study are often selected on the basis of annotation and therefore could produce biased results. Two recently published databases of GWAS hits ([15] [16], hereafter referred to as ‘Hindorff’ and ‘Johnson’) have provided the necessary resources to carry out such an investigation. Both contain >2000 SNPs with a low p-value for association in at least one GWAS, although there are several differences between the datasets which are discussed below. Both groups performed some analysis of the data. Hindorff et al analysed hits with a p-value <5×10^−8^ whereas Johnson and O’Donnell analysed all the results in their dataset (p-values <0.05) [7], [16]. Hindorff et al looked at 20 different annotations and established that non-synonymous sites and 5kb promoter regions are enriched in GWAS hits relative to regular GWAS panel SNPs. Johnson and O’Donnell demonstrated that SNPs that are hits in multiple studies are more likely to be true hits. They also described an over representation of hits in genes related to cell adhesion functions. More recently Nicolae et al [17] have established an overrepresentation of expression QTLs (eQTLs) in the Hindorff database.

It is not clear how dataset-specific these previous findings might be. In this paper, we compare and contrast two GWAS hit datasets and perform sensitivity analysis to gauge the robustness of annotation enrichment under different conditions. We focus on three annotations from three different categories, non-synonymous SNPs (nsSNPs), promoter SNPs and *cis* expression QTLs (eQTLs) lying in open chromatin regions, representing three major classes of annotation information: protein changes, gene regulation and gene expression. We determine if these annotations are enriched across both datasets carrying out some additional analysis to verify the robustness of the findings. We find that Hindorff’s results in relation to nsSNPs and promoter SNPs and Nicolae’s results relating to eQTLs are broadly repeated across both datasets. We show how these findings can be built into a Bayesian analysis of association results.

## Methods

### GWAS hit SNPs

We used two published GWAS datasets: ‘Hindorff’ ([15]) and ‘Johnson’ ([16]). Both datasets were compiled using literature searches of Pubmed and other sources. The Hindorff dataset has a p-value cut-off of 10^−5^ (although Hindorff et al [7] only performed analyses on SNPs with a p-value less than 10^−8^), while the Johnson dataset uses a p-value cut-off of 0.05. The Hindorff dataset is continually updated whereas the Johnson one is not. The latter includes all GWAS published up until 1^st^ March 2008. We downloaded the Hindorff dataset on the 21^st^ May 2010, at which point it contained 2727 unique SNPs with results for single marker analyses. The Johnson dataset contained 52546 SNPs, but we performed most of our analyses on data with p-values less than 10^−5^ (4086 SNPs). After noting a large excess of SNPs in the major histo-compatibility (MHC) region in the Johnson dataset we filtered out SNPs from this region (chr6:25809985-33486934) in both datasets, as results from this region could be unrepresentative of results throughout the rest of the genome due to the high density of genes and extensive long range linkage disequilibrium. This left 2115 unique SNPs from 425 studies in the Hindorff dataset and 2695 from 96 studies in the Johnson dataset (constrained to p<10^−5^). We also filtered out hit SNPs that were not on the original GWAS panels as they are often selected on the basis of annotation to support the replication. Except where otherwise stated all results presented relate to this subset of the data.

### GWAS panel SNPs

We adopted a sensitivity analysis approach in which we contrasted results obtained under two very different scenarios, representing two extreme possible endpoints of average GWAS panel SNP composition. In one we assumed all GWASs had used the Affymetrix Mapping 500K panel (hereafter ‘Affy500’) and in the other that all GWASs had used the Illumina HumanHap 550K panel (hereafter ‘Illu550’). Both panels have been widely used in GWASs to date, but reflect different strategies for marker selection. Illumina selected tagging SNPs whereas Affymetrix selected SNPs based on assay availability and minor allele frequency. The proportion of SNPs with a MAF less than 0.1 on the Illu550 is 22% whereas the proportion on the Affy500 is 34%. In addition to these two extreme approaches, we considered a compromise GWAS panel set comprising the union of these two panels (hereafter ‘Affy500+Illu550’).

### Annotation

We chose three annotation categories; non-synonymous SNPs (nsSNPs), expression quantitative trait loci (eQTLs) and promoter region SNPs. Non-synonymous SNPs alter the amino acid sequence of a gene product, we downloaded these from the UCSC browser selecting nonsense (premature termination codons) and missense mutations from the dbSNP version 130 table.

eQTLs are excellent candidates for GWAS hits as they are thought to be causally involved in complex traits and may be more closely correlated to the genotype than the complex trait itself. We defined and selected eQTLs from a study of global gene expression in lymphoblast cell lines (LCLs) [18]. Some 55,000 transcripts representing 21,000 genes were investigated and approximately 15,000 transcripts (from 7,000 genes) demonstrated heritability. These transcripts were tested as a GWAS and all SNP-transcript pairs with regression p-values <0.001 were retained. We defined eQTLs based on the rank of this p-value. In one set of analyses we used a stringent cut-off, defining eQTLs as only those that had a p-value in the top 20,000. For the other set of analysis we used p-values in the top 100,000. We performed analysis on all eQTLs and also on *cis*-eQTLs (those within 200 kb of the transcript they are associated with). These selection criteria allowed us to explore a number of approaches to defining eQTLs. We also identified eQTLs within open chromatin regions as these are much more likely to be involved in regulation of expression. We used evidence of open chromatin in multiple cell lines from the Duke/UNC/UT-Austin/EBI ENCODE group made available on UCSC. Open chromatin regions were identified using two independent and complementary methods: DNaseI hypersensitivity (HS) and Formaldehyde-Assisted Isolation of Regulatory Elements (FAIRE), combined with chromatin immunoprecipitation (ChIP) for selected regulatory proteins. Each method was verified by two detection platforms: Illumina (formerly Solexa) sequencing by synthesis, and high-resolution 1% ENCODE tiled microarrays supplied by NimbleGen.

We used the First exon finder (firstEF) program to identify putative promoter regions, defined as the 570 bp immediately upstream of the first exon [19]. Many genes have completely non-coding first exons (i.e. fall entirely within the 5’ UTR). It is therefore important to check that the reported first exon for a gene does not have an upstream splice donor as this would suggest that the true first exon (and promoter region) has not been correctly identified. FirstEF identifies splice donor sites and uses discriminant functions to identify true first exons and their promoters regions. We ran FirstEF on the hg18 (build 36) table within the UCSC genome browser, it identified 74737 promoters (many more than the number of putative genes because many genes have alternative promoters and alternative first exons).

Our approach to testing for annotation enrichment was to compare the proportion of annotated SNPs in the GWAS hit SNP sets with the GWAS panel SNP sets. We determined standard error bars and statistical significance based on expected binomial variation in the GWAS hits (as the number of SNPs in different annotation classes in the GWAS panel sets was large enough to result in negligible error by comparison).

### Linkage disequilibrium (LD) proxies

GWAS panels do not include every SNP in the genome, and it is expected that many GWAS hits will only be markers for true causal variants, lying outside the GWAS panel, that are associated via linkage disequilibrium or ‘tagging’. We address this issue by annotating our GWAS SNPs (both ‘hits’ and ‘nulls’) via LD-proxy. A SNP was defined to be LD-proxy-annotated if it was in linkage disequilibrium with an annotated SNP with r^2^> = 0.8. We used the SNAP web-tool [20] to determine the LD proxies for all GWAS SNPs, based on the HapMap Phase 2 CEU reference population [21]. This population was chosen because most GWAS studies in both datasets are largely made up of Caucasian individuals.

We note that eQTL annotations already have an element of linkage disequilibrium ‘built in’, as any SNP labelled an eQTL may itself be only tagging a nearby causal SNP. However, our eQTL dataset derives from a smaller GWAS panel (Illumina 300k), making further extension via LD-proxy necessary.

### Bayes Factors for Bayesian analysis

Bayesian analysis provides the most suitable framework for combining annotation information with evidence from an association study [22]. The posterior odds (O_post_) of true association (meaning a direct or indirect causal effect) for the trait of interest at a given SNP are defined as the ratio of the conditional probability of causality, given the annotation and association data, to the conditional probability of non-causality:
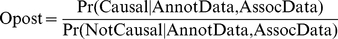
This quantity can be found as the product of the following ratios (given that the annotation data and association data are independent once conditioned on causality):
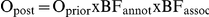
Where O_prior_ are the prior odds before seeing any data, thus O_prior_  =  Pr(Causal)/Pr(Not Causal); BF_annot_ is the Bayes Factor for the annotation data, thus BF_annot_  =  Pr(Annot Data | Causal)/Pr (Annot Data | Not Causal); and BF_assoc_ is the Bayes Factor for the association data, thus BF_assoc_  =  Pr(Assoc Data | Causal)/Pr (Assoc Data | Not Causal).

Note that our definition of ‘true association’ includes the possibility of indirect association via linkage disequilibrium. To account for this, we import annotation data from other SNPs in LD, as we describe above. We also note that BF_assoc_ will typically refer to a hypothesis of causality for a specific phenotype, whereas the BF_annot_ values that we consider below refer to a hypothesis of causality for any phenotype that has been tested in a GWAS. Our method is therefore motivated by the idea that the BF_annot_ values obtained under a general-phenotype definition of causality are a reasonable guide to the BF_annot_ values one would obtain for the specific phenotype in question.

The prior odds, O_prior_, are set in advance, and are usually set to reflect a low prior belief that any one given SNP in the human genome is causally related to the phenotype in question (as indeed reflected by the small number of GWAS hits found so far for most complex traits). For example, O_prior_  = 10^−5^ was used by the Welcome Trust Case Control Consortium [2]. In cases where only the relative ranking of SNPs is of interest (for example, where a fixed number of SNPs to be taken forward for follow-up), then the value of O_prior_ is unimportant as it will not affect the relative rankings of O_post_.

The Bayes Factor for association, BF_assoc_, is calculable from GWAS data either via direct computation of the relevant integral [2] or via an approximation which removes the need for integration [23].

The Bayes Factor for annotation, BF_annot_ is estimated empirically from the GWAS hit data. The estimated value is the proportion of a given annotation class seen in the set of hit SNPs divided by the proportion seen in the set of non-hit SNPs. Since hit SNPs make up a small fraction of all SNPs, we shall use the annotation proportion seen in unselected GWAS panel sets for this latter quantity.

### Application to real data

Application of our method to real data would require the following steps: (1) decide on prior odds (if absolute rather than relative O_post_ values are required); (2) calculate BF_assoc_ from GWAS data; (3) calculate BF_annot_ from GWAS hit database data; (4) calculate posterior odds using the formula given above. To facilitate our method, we have made available software for calculating BF_assoc_ from PLINK output files, and have created a file containing BF_annot_ values for all the SNPs on the Affy500 and the Illu550 panels, indicating their annotation status for the three categories under study as well as BF_annot_ in the range that we recommend using. These resources are available from our website: http://www.kcl.ac.uk/schools/medicine/research/genetics/research/clusters/bse/weale/software.

We tested our method on a real dataset. We compared the rank of the BF_assoc_ with the rank of the product of the BF_assoc_ and the BF_annot_ in the WTCCC1 Crohn’s data[2]. We determined the changes in rank of the 48 loci that have been recently determined to be involved in the trait only 9 of which were demonstrated to be strongly associated with the trait in the WTCCC1 study [24]. (Only 48 of the recently published 71 were used because the others were neither present nor represented by proxies on the Affy500 or Illu550 panels.) We also determined the rank change for 100 sets of 48 randomly selected SNPs. We used the Wakefield method to derive the BF_assoc_ [23].

## Results

### General annotation enrichment and sensitivity analyses

**Figure 1:**
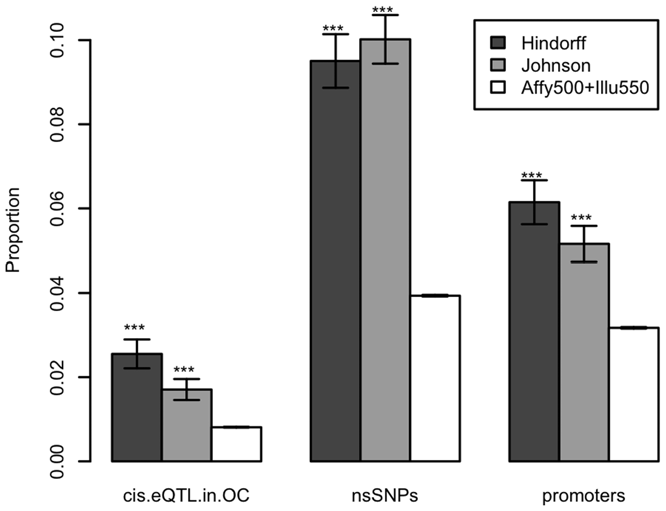
Annotation proportions in the Hindorff and Johnson GWAS hit datasets and a GWAS panel set. The proportion of annotation is shown for three different categories (cis eQTL in open chromatin, nsSNPs and promoter SNPs). “***” indicates p-values <2.8×10^−5^ ( = 0.001/36); “**” indicates p-values <2.8×10^−4^ ( = 0.01/36); “*” indicates p-values <1.4×10^−3^ ( = 0.05/36). These thresholds were chosen to reflect a Bonferroni correction of the 36 comparison tests implicit in Figures 1 and 2. The error bars represent the standard error of the estimated proportions (normal approximation to binomial distribution). The GWAS panel set is comprised of a union of Affymetrix 500k and Illumina 550k panels.

A higher proportion of SNPs have functional annotation in the GWAS hit datasets compared to the GWAS panel SNPs (Figure 1 and Table 1). The p-values for all these differences were less than 2.8×10^−5^ which is equivalent to a level of 0.001 after Bonferroni adjustment for multiple testing.

**Table 1:** GWAS SNP set annotation counts (with percentage of total in brackets).

	Hindorff	Johnson	Affy500+Illu550
Total (hit SNPs: P<10^−6^)	1219	1576	961605
cis eQTLs in Open Chromatin	46 (3.8)	39 (2.5)	7791 (0.8)
ns SNPs	166 (13.6)	181 (11.5)	37856 (3.9)
promoter SNPs	97 (8)	89 (5.6)	30516 (3.2)
No annotation	1853 (87.6)	2380 (88.3)	908537(94.5)

For the GWAS hit SNP datasets, the number of SNPs with p-values <10^−6^ that fall into each annotation categories is presented. SNPs in each annotation categories include annotated SNPs and their linkage disequlibrium proxies.

**Figure 2:**
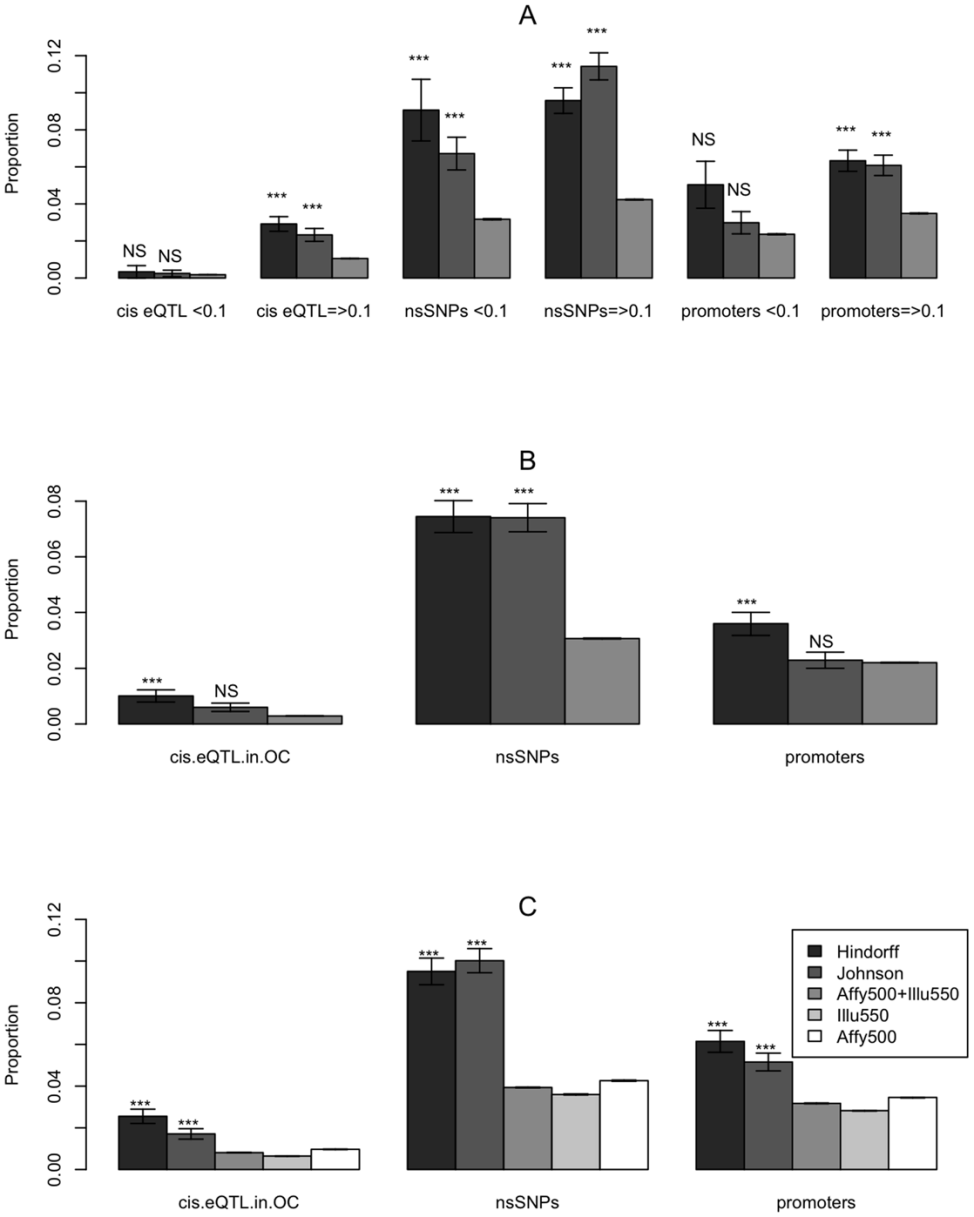
Annotation proportions of subsets the hit datasets and a selection of GWAS panel sets. The proportion of annotation is shown for three different categories (cis eQTL in open chromatin, nsSNPs and promoter SNPs). Significance levels and error bars are defined as in Figure 1. Panel A is stratified by minor allele frequency, panel B contains only SNPs with unique annotations and panel C compares different GWAS panels.

We observed that the Hindorff dataset had 13.6% of SNPs with MAF<0.1, while the Johnson dataset had 29.8% of SNPs with MAF<0.1, a similar figure was seen in the Affy500+Illu550 panel (27.9%). This bias may be due to the fact that the Hindorff dataset often only contains the most significant SNP in a region. It is likely that such SNPs will have a relatively high MAF compared to others in the region as it is hard for SNPs with very low MAF to attain small p-values. To test the results for robustness against the differences in MAF distributions, the proportion of annotation was compared for SNPs with MAF <0.1 and SNPs with MAF  = >0.1 (Figure 2, panel A). The proportion of annotation was again found to be lower in the Affy500+Illu550 panel than in either GWAS hit datasets for all annotations. As the pattern with respect to the GWAS panel SNPs was generally consistent across the MAF range, we performed further analyses on the complete datasets irrespective of MAF.

To establish whether the results from the three categories were independent, we removed all SNPs that had a multiple annotation or were a proxy for any SNP in another annotation category. The patterns remained consistent (Figure 2, panel B). The remaining analyses were performed on all SNPs, including those with multiple annotations.

To investigate whether the results were specific to the chosen ‘null’ GWAS panel set, we compared the annotation proportions seen in the Affy500-only and Illu550-only GWAS panels. Since different SNP selection strategies were adopted by Affymetrix and Illumina in constructing their panels, and in particular in the SNP tagging approach used by Illumina, splitting the GWAS panel dataset in this way allowed us to perform a sensitivity analysis with respect to the different SNP selection strategies and their effect on GWAS panel composition. We found consistently lower proportions of annotation in all three GWAS panel sets, compared to either GWAS hit sets (Figure 2, panel C). We therefore performed further analyses using the combined Affy500+Illu550 set.

### Estimating Bayes Factors

We suggest the use of Bayes Factors of 0.93 for SNPs without annotation and within the range of 3.1–4.7 for *cis* eQTLs in open chromatin, 2.9–3.5 for nsSNPs, 1.8–2.5 for promoter SNPs. These ranges take into account the results from both datasets when proxies of the annotated SNPs with r^2^s of > = 0.8 are included.

**Figure 3:**
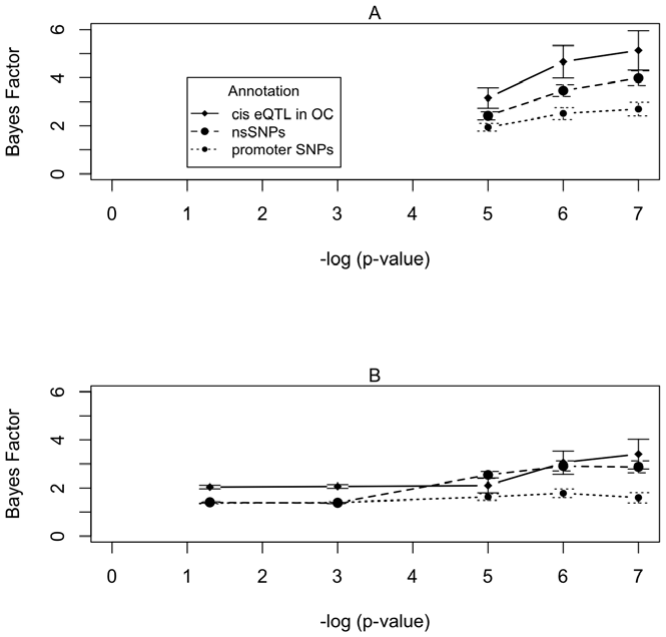
Bayes Factors estimated from GWAS hit sets defined using a range of p-value cut-offs. The Bayes Factors are shown for three different categories (cis eQTL in open chromatin, nsSNPs and promoter SNPs). Panel A shows results derived using the Hindorff dataset and Panel B results from the Johnson dataset. The GWAS panel set is comprised of a union of Affymetrix 500k and Illumina 550k panels.

Figure 3 shows that when more stringent p-value thresholds are used to define GWAS hits, the Bayes Factors increase. This provides further evidence that annotation enrichment is not due to some artefact, as this pattern is consistent with the proportion of true GWAS hits increasing as p-value stringency increases. We consider Bayes Factors calculated at the p-value cut-off of 10^−6^ to be the most appropriate for use. This p-value cut-off balances the requirement for stringency that will enrich for selection of true hits and lenience to ensure enough SNPs are included to allow a reasonably accurate measure of the Bayes Factor.

### Linkage disequilibrium proxies

We use ‘LD-proxy-annotations’ (see Methods) to address the issue that many GWAS hits will not be directly causal, but will only tag an off-panel causal variant by linkage disequilibrium. However, our method relies on an arbitrary threshold (r^2^> = 0.8). We therefore performed sensitivity analyses on the effect of LD proxy threshold.

**Figure 4:**
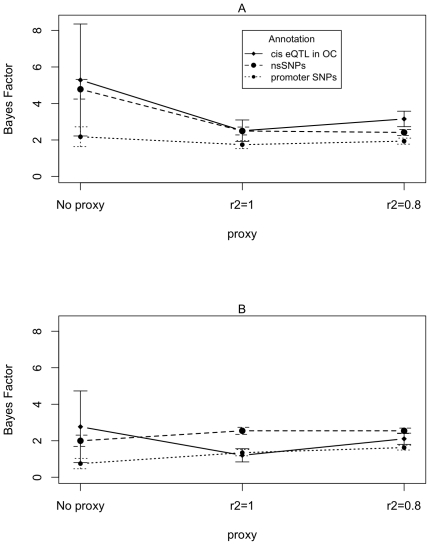
Bayes Factors estimated with and without linkage disequilibrium proxies for annotated SNPs. The Bayes Factors are shown for three different categories (cis eQTL in open chromatin, nsSNPs and promoter SNPs). Panel A shows results derived using the Hindorff dataset and Panel B results from the Johnson dataset. The GWAS panel set is comprised of a union of Affymetrix 500k and Illumina 550k panels.

We performed most analysis using proxies with an r^2^ of > = 0.8 and tested the effect of this cut-off by performing analysis using proxies with an r^2^ of 1, and analyses with no proxies at all. The variation in threshold did not have much of an impact on the results (Figure 4). There is some variation in Bayes Factors, but there is no evidence that those calculated using LD proxies are systematically biased.

### eQTL definition

**Figure 5:**
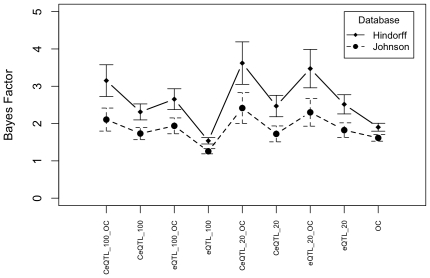
Estimated Bayes Factors for alternative eQTL definitions. The GWAS panel set is comprised of a union of Affymetrix 500k and Illumina 550k panels.

In our preliminary analysis we investigated *cis* eQTLs in open chromatin only selecting the SNPs that had a p-value ranked in the most significant 100,000. However we also calculated Bayes Factors for both *cis* and *trans* eQTLs and for eQTLs with a p-value ranked in the most significant 20,000. For each category we also calculated Bayes Factors for all SNPs as well as only for those SNPs in open chromatin (Figure 5).

In each direct comparison the SNPs in open chromatin had the greater Bayes Factor. The most highly significant cis eQTL category had the greatest Bayes Factor. The increase in stringency and selection of only *cis* eQTLs both increase the Bayes Factor but it is important to note that these are not independent selection criteria. When the top 20,000 eQTLs are selected 74.9% of these are *cis*, when the top 100,000 are selected only 30.3% of these are *cis*.

### Application to real data

The rank of the BF_assoc_ * BF_annot_ was on average 10322 higher than the rank of the BF_assoc_ for the Crohn’s hits and 205 lower for the null hits. Furthermore 21 of the Crohn’s hits moved up in rank while the average number that moved up in the null set was only 4.

## Discussion

Our study confirms the hypothesis that there are differences in the proportion of functional annotation between GWAS hits and the background of GWAS panel SNPs. This trend is robust to differences in GWAS panel SNP sets, different GWAS hit lists and SNP allele frequency. The patterns are also independently seen in each annotation category. This provides us with reassurance, given the problems experienced both with accurately capturing all GWAS hits and with defining a fully appropriate comparative GWAS panel set. Our study highlights three categories of functional annotation that appear to provide reliable enrichment in GWAS data that can be used to empirically estimate Bayes Factors for Bayesian analysis. Furthermore when applied to real data our technique increases the rank of SNPs that have later been shown to be hits.

In order to produce hit SNPs sets with reasonably large numbers of SNPs, our definition of a GWAS ‘hit’ includes SNPs with p-values greater than what is typically considered to be genomewide significant. We accept that this increases the proportion of false positives in our hit sets. However, our sensitivity analyses show that annotation enrichment is still noticeable in hit SNP sets with a lower p-value threshold definition. We also note that the overall effect of false positives in the set of GWAS hits will be to shrink BF_annot_ values towards 1, so it will have a conservative effect on the use of annotation information in combination with association data.

The robustness of these results across the datasets and indeed the different ways of defining annotations and GWAS hits is striking, particularly in relation to the eQTLs. The eQTLs in our study and Nicolae et al’s [17] were both defined from lymphoblast cell lines, but the eQTL dataset we used was defined in families ascertained on the basis of a proband with asthma [18] whereas Nicolae et al defined eQTLs using HapMap individuals. Nicolae et al used a p-value cut-off which led them to define 40% of the Hindorff dataset as eQTLs whereas we used a ranking system that identified 2.5% of the Hindorff dataset as eQTLs. Furthermore Nicolae et al controlled for MAF by sampling null SNPs with matching MAF rather than comparing annotation within different bins. Despite these differences in data and study design eQTL enrichment is evident across both studies.

While the patterns of enrichment are broadly consistent, our study also reveals some differences. The annotation proportions, and derived Bayes Factors, from the Hindorff dataset are almost always higher than from the Johnson dataset. There is also a difference in the ranking of the three categories, in the Hindorff dataset *cis* eQTLs always have the highest Bayes Factor and promoter SNPs the lowest. This is the case in most but not all of the analysis on the Johnson dataset. This reflects ascertainment differences between the two datasets. One notable difference is the number of SNPs included per study, with Johnson including 28 on average and Hindorff only 5. This can be linked to a number of factors. When Hindorff et al began collating their dataset they only included one SNP in each associated region whereas the Johnson dataset include all of them. The Johnson dataset also included more hits where the information came from supplementary tables and/or was derived from an alternate statistical test. The Bayes Factors are also affected to some extent by the choice of reference GWAS panel, by the inclusion or exclusion of LD proxies, and by the choice of p-value threshold used to define GWAS hits.

It is not straightforward to arrive at an appropriate ‘null’ set of GWAS SNPs, against which the annotation properties of a hit set can be compared. For example, consider combining the results of one GWAS that used the Affymetrix GeneChip Human Mapping 500K panel with another that used the Illumina HumanHap 550K panel. These panels share about 15% of SNPs. Should the annotation information for these SNPs held in common be counted twice (summation approach) or only once (union approach)? Our null hypothesis is not that all these GWAS hits are false (we assume in fact that most are true), but rather that their location is independent of any annotation information that may be attached to them. The summation approach is appropriate if we assume that the GWAS hits in the second study are independent of the first study (e.g. unconnected diseases, no common causal genetic mechanisms), while the union approach is appropriate if the same hits are to be expected (e.g. same or very similar disease, with both studies well powered). Given that both datasets contain several GWASs on the same or similar phenotypes, and given the growing evidence for some causal effects spanning many diseases, the best situation would lie somewhere between the two approaches. In addition to this theoretical uncertainly, there is also considerable practical uncertainty in ascertaining exactly which panels were used in each study, especially in studies where more than one panel was used. Even if the panels are known, the set of SNPs remaining after QC may not be. The panel composition of each GWAS study is important because there are between-panel differences in the selection strategies for panel membership, based on features such as minor allele frequency, linkage disequilibrium and location (e.g. genic vs inter-genic), and all of these may impact on the annotation proportions. Again the consistency of results accross panels demonstrates the validity of the approach despite these problems.

We accept that it will be difficult to determine exact values for empirically derived Bayes Factors. However, there is sufficient consistency in our study for us to suggest the use of Bayes Factors within the range of 3.1–4.7 for *cis* eQTLs in open chromatin, 2.9–3.5 for nsSNPs and 1.8–2.5 for promoter SNPs. If an investigator chooses to increase weightings on the annotations they would use the weight at the top of the range, if they wanted to limit the influence of the annotation they would use a weight from the bottom of the range. In those cases where more than one annotation is attached to a SNP, either directly or via LD proxy, our datasets are not large enough to present direct empirical answers. We propose conservatively that the annotation with the largest Bayes Factor be used in such cases, on the assumption that a second observed annotation may increase but never decrease the Bayes Factor of the first annotation.

We allowed GWAS panel and GWAS hit SNPs to acquire “annotation-via-LD-proxy”, primarily because GWAS panel are designed to detect association signals via tagging. In addition to this the use of proxies increases the size of the datasets that we are working with. An alternate approach would have been to amplify the set of SNPs to include all LD proxies of all GWAS panel SNPs, and indentify “hits-by-proxy” and “nulls-by-proxy”. However, under this approach is is not clear what to do with SNPs which are simultaneously “hits-by-proxy” and “nulls-by-proxy”, a problem which is avoided by our approach.

In this study we have not differentiated GWAS hits by phenotype, both because we are interested in general determinants of causality and because stratifying the GWAS hits in this way decreases the power to identify differences in the distributions of the annotation between the datasets. However, we note that using their alternative approach of defining eQTLs, Nicolae et al [17] found that the enrichment was present across a number of different phenotype classes, even those in which you would not expect expression in the lymphoblast cell lines to play a role in the disorder.

Due to advances in next generation sequencing technology [25], large amounts of sequence variant data are now becoming available, particularly focused on the discovery of rare pathogenic variants. Bayes Factors can also be used to prioritise hits from such datasets for follow up. In time, Bayes Factors will need to be derived on the basis of the results of sequencing experiments as these become public. In the interim, we note that MAF does not appear to have a large influence on our estimated Bayes Factors from GWAS data (Figure 2), which presents the possibility of using the same Bayes Factors estimated here from GWAS data in sequence analysis, until such time as enough relevant sequence data becomes available.

The enrichment signal found in this study for different functional annotation categories in GWAS hits is sufficiently consistent, and the size of the enrichment sufficiently large, to justify its use in Bayesian association analyses. More work is needed to define the size of the signals in other annotation categories, and to refine how rare variants identified by next generation sequencing differ from common variants identified in GWAS data.
